# Linoleyl acetate and mandenol alleviate HUA‐induced ED via NLRP3 inflammasome and JAK2/STAT3 signalling conduction in rats

**DOI:** 10.1111/jcmm.70075

**Published:** 2024-09-08

**Authors:** Pingyu Ge, Hong Xie, Yinxue Guo, Hang Jin, Lan Chen, Zhichao Chen, Yan Liu

**Affiliations:** ^1^ First Clinical College of Guizhou University of Traditional Chinese Medicine Guizhou Province China; ^2^ Department of Urology Surgery First Affiliated Hospital of Guizhou University of Traditional Chinese Medicine Guizhou Province China; ^3^ Department of Nephrology The First Affiliated Hospital of Guizhou University of Traditional Chinese Medicine Guizhou Province China; ^4^ Department of Cardiology Qilu Hospital of Shandong University Shandong Province China

**Keywords:** erectile dysfunction, hyperuricemia, linoleyl acetate, mandenol, NLRP3 inflammasome

## Abstract

Hyperuricemia (HUA) is characterized by elevated blood uric acid levels, which can increase the risk of erectile dysfunction (ED). Clinical studies have demonstrated satisfactory efficacy of a traditional Chinese medicine formula QYHT decoction in improving ED. Furthermore, the main monomeric components of this formula, linoleyl acetate and mandenol, demonstrate promise in the treatment of ED. This study established an ED rat model induced by HUA and the animals were administered with linoleyl acetate and mandenol. HE and TUNEL were performed to detect tissue changes, ELISA to measure the levels of serum testosterone (T), MDA, NO, CRP, and TNF‐α and qPCR and WB to assess the expression levels of NLRP3, ASC, Caspase‐1, JAK2, and STAT3 in whole blood. The findings showed that linoleyl acetate and mandenol improved kidney tissue morphology, reduced cell apoptosis in penile tissue, significantly increased T and NO levels, while substantially decreasing levels of MDA, CRP, and TNF‐α. Meanwhile, the expression of NLRP3, ASC, and Caspase‐1 mRNAs and proteins was markedly reduced, and the phosphorylation of JAK2 and STAT3 was inhibited. These findings were further validated through faecal microbiota transplantation results. Taken together, linoleyl acetate and mandenol could inhibit NLRP3 inflammasome activation, reduce inflammatory and oxidative stress responses, suppress the activity of JAK–STAT signalling pathway, ultimately providing a potential treatment for HUA‐induced ED.

## 1 INTRODUCTION

Hyperuricemia (HUA) represents a metabolic disorder by dysregulation in purine metabolism, resulting in either excessive production or reduced excretion of uric acid, thereby leading to elevated blood uric acid levels.^1^ It is clinically defined as serum uric acid levels equal to or greater than 420 μmol/L in males and 360 μmol/L in females.^2^ Within normal ranges, blood uric acid levels exhibit anti‐inflammatory, antioxidant and vasodilatory properties. Being elevated in the bloodstream, it can trigger metabolic disorders and contribute to the development of various diseases. In recent years, shifts in lifestyle and dietary patterns have led to a gradual increase in the prevalence of HUA,^3^ significantly impacting public health. In medicine field, uric acid synthesis inhibitors are commonly used to control uric acid levels and alleviate the discomfort and complications associated with HUA. Erectile dysfunction (ED) is a common male sexual disorder that can arise from various factors. It is characterized by the inability of the male to achieve or sustain a sufficient erection for satisfactory sexual intercourse within the preceding 3 months. Statistical data indicate that the risk of developing ED increases gradually with age, particularly among males aged 80 years and older, with a prevalence rate as high as 86%. It is estimated that by 2025, global population of individuals with ED will reach 322 million.^4^ Consequently, the management of ED is of important clinical significance and warrants further investigation.

A 10‐year UK prospective cohort study enrolled 50,000 participants has reported a significantly increased risk of ED in all patients with HUA.^5^ Another case–control study by Salem et al.^6^ has revealed that blood uric acid levels in ED patients were significantly higher than those in non‐ED patients, and they also noted that with every 1 mg/dL increase in serum uric acid level, the risk of developing ED doubled, thus reinforcing the recognition of HUA as a distinct risk factor for ED. Other studies have suggested that HUA may lead to endothelial dysfunction, reducing the bioavailability of nitric oxide (NO), and inhibiting vasodilation. Since vascular endothelial dysfunction is a primary mechanism underlying ED, these findings highlight the pathophysiological correlation between HUA and ED.^7^ However, the mechanism linking HUA to ED is not clear at present. Some studies speculate that HUA may induce oxidative stress and tissue damage by accelerating the release of reactive oxygen species from vascular endothelial cells.^8^ Alternatively, it is proposed that HUA can inhibit testosterone levels, leading to male hypogonadism and promoting the development of ED.^9^ On the other hand, HUA assumes a pivotal role in apoptosis pathway by stimulating the expression and release of inflammatory cytokines through a series of complex mechanisms.^10^ These results provide valuable insights into the mechanism of HUA.

Previous experiments have showed that administering a traditional Chinese medicine formula, QYHT decoction, to HUA patients can significantly improve both ED and vascular endothelial cell function, yielding a satisfactory clinical effect.^11^ QYHT decoction consists of *Semen Coicis*, *Plantaginis Semen*, *Fritillariae Thunbergii Bulbus*, *Pheretima*, *Epimedii Herba* and *Curculiginis Rhizoma*. This decoction has the effect of removing phlegm and blood stasis, relieving the internal resistance of phlegm dampness, and aiding in the promotion of blood circulation of patients, thereby reducing the impact on vital organs such as the kidneys and lowering the occurrence of impotence and related complications. Among the components, linoleyl acetate, the main monomeric component in *Pheretima*, *Epimedii Herba*, plays a critical role in the treatment of polycystic ovary syndrome^12^ and mandenol is the major monoer of *Semen Coicis*. Zhang et al.^13^ have found that mandenol can treat gout by targeting the Hippo signalling pathway via CTNNB1 protein. Gout is the most common inflammatory arthritis caused by HUA, which leads to the deposition of from uridate crystals in joints, tendons, and other tissues,^14^ and these findings suggest that mandenol may be a therapeutic treatment for HUA. The bioactive chemical compounds in traditional herbal medicine have been proven to have potential for use as therapeutic agents.^15^ Linoleyl acetate and mandenol, as important compounds of the QYHT decoction, may play a significant role in the treatment of ED. However, existing studies have provided limited reports on the effects of linoleyl acetate and mandenol on HUA. Therefore, in this study, a rat model of HUA‐induced ED was established to explore whether linoleyl acetate and mandenol have therapeutic potential for HUA‐induced ED. Intensive investigation of these monomers may yield crucial insights for the development of new therapeutic strategies.

## 2 MATERIALS AND METHODS

### 2.1 Establishment of HUA‐induced ED rat models

In our study involving mandenol, 60 male SD rats, aged 6–8 weeks, were randomly divided into five groups using a completely random method, with each group consisting of 12 rats. One group served as the control, while the remaining four groups were used for model establishment. During the feeding period, yeast powder was added at a concentration of 15 g/kg. Additionally, to induce the model, the rats received an intragastric administration of an ethyl carbamate suspension (250 mg/kg body weight) and subcutaneous injections of potassium oxonate (200 mg/kg body weight). The control group received a regular diet and was treated with an equivalent volume of physiological saline.

The model establishment period lasted for 8 weeks. On the 57th day, blood samples were collected from the rats' tail veins and centrifuged at 4000 r/min for 15 min to extract the serum. Uric acid (UA), blood urea nitrogen (BUN), and creatinine (Cr) levels were determined using an AMS‐18 automatic analyser to confirm the successful establishment of the HUA model. Subsequently, rats with HUA‐induced ED were selected and placed in an observation cage with subdued lighting to acclimate for 10 min. These rats were then administered a subcutaneous injection of apomorphine (APO, 100 mg/kg) and observed for 30 min. Rats exhibiting penile erection were classified as APO (+) HUA rats, whereas those without penile erection were classified as HUA‐induced ED rats.

The experiment was divided into five groups: control, model, low‐dose mandenol (L‐mandenol) group, medium‐dose mandenol (M‐mandenol) group and high‐dose mandenol (H‐mandenol) group. The control group was maintained under routine culture conditions. The model group consisted of HUA‐induced ED rats. The L‐mandenol group received 8 mg/kg mandenol, the M‐mandenol group received 16 mg/kg mandenol, and the H‐mandenol group received 32 mg/kg mandenol, each based on the model group configuration. Eleven weeks later, the experiment was conducted. The linoleyl acetate groups were treated with doses of 40, 80, and 160 mg/kg, respectively, using the same methods described above.

### 2.2 HE staining

After obtaining renal tissues from male rats, the procedure began with PBS rinsing. Subsequently, the specimens were fixed in a 4% paraformaldehyde solution and immersed separately in 75% ethanol, 85% ethanol, 95% ethanol, 100% ethanol I, and 100% ethanol II for a duration of 2 h each. After dehydration, the tissues were sequentially immersed in 1/2 xylene, xylene I, and xylene II for 10 min each, followed by embedding in molten paraffin to produce 2.5 μm‐thick sections. These sections underwent deparaffinization and rehydration, followed by rinsing with dual distilled water, and 35‐min staining using Servicebio's G1004 haematoxylin. After decolorization, a 2‐min staining with eosin dye was conducted, followed by thorough rinsing to eliminate excess dye. After dehydration, we performed transparency using xylene and sealed the sections with neutral resin (10004160, Sinopharm, Beijing, China). The final step involved examination under a microscope, where cell nuclei appeared blue, and the cytoplasm exhibited red or pink shades. Throughout this process, a Mshot MF53 microscope from Guangzhou Mshot Technology Co., Ltd. was used to capture images.

### 2.3 TUNEL staining

After preparation, the penile tissues were performed dewaxing and rehydration, delicately rinsed with PBS, treated with an appropriate amount of 20 μg/mL proteinase K working solution, and incubated at 37°C. Subsequently, the cells were washed with PBS. Under the room‐temperature processing, an adequate amount of 3% H_2_O_2_ (prepared in PBS) was added to the tissues, which were then washed with PBS, added with an appropriate amount of Equilibration Buffer, allowing to cover the area of the sample to be examined, and incubated at room temperature. Each tissue sample was supplied with an adequate amount of TdT incubation buffer, following the ratio of Recombinant TdT enzyme to Biotin‐dUTP Labeling Mix to Equilibration Buffer as 1:5:50, and was incubated at 37°C. Subsequently, the tissues were promptly rinsed with PBS. Each sample tissue was treated with a diluted streptavidin‐HRP reaction solution (Streptavidin‐HRP to TBST ratio of 1:500) and incubated at 37°C. Following a PBS wash, an adequate volume of DAB staining working solution was applied for subsequent microscopic examination after sealing the sections. The results revealed that apoptotic cell nuclei with positive staining exhibited a brownish‐yellow colour, whereas apoptotic cell nuclei with negative staining appeared blue.

### 2.4 Serum ELISA

The serum levels of NO, testosterone (T), malondialdehyde (MDA), C‐reactive protein (CRP) and tumour necrosis factor‐alpha (TNF‐α) were determined using assay kits from mlbio (Shanghai, China) for NO and from RayBiotech (Guangzhou, China) for T, MDA, CRP, and TNF‐α, following the respective kit instructions.

### 2.5 qPCR

Extraction of blood RNA was performed following the detailed instructions of the PureLink Total RNA Blood Purification Kit (Invitrogen, Carlsbad, CA, USA). The PrimeScript™ II 1st Strand cDNA Synthesis Kit (Takara, Tokyo, Japan) was used for cDNA synthesis. For qPCR, the reaction system was prepared with the TB Green® Premix Ex Taq™ II kit and amplification was carried out using the CFX Connect instrument (Bio‐Rad Laboratories, Hercules, CA, USA). GAPDH was chosen as the reference gene for data normalization.

The primers utilized in this study were presented as follows:

NLRP3 (forward): 5′‐CCGTCGTCTTTGAGCCTTCT‐3′,
NLRP3 (reverse): 5′‐GGATGGATCGCAGCTCTCTC‐3′;
ASC (forward): 5′‐ATCCAGGCCCCTCCTCAG‐3′,
ASC (reverse): 5′‐AGAGCTTCCGCATCTTGCTT‐3′;
Caspase‐1 (forward): 5′‐GTGCAGGACAACCCAGCTAT‐3′,
Caspase‐1 (reverse): 5′‐CGTGCTGTCAGAGGTCTTGT‐3′;
JAK2 (forward): 5′‐AAGGATCTGGTATCCACCCA‐3′,
JAK2 (reverse): 5′‐CCCACGAGATACTCCGTACC‐3′;
STAT3 (forward): 5′‐GGAGGAGGCATTCGGAAAGT‐3′,
STAT3 (reverse): 5′‐TCCAAACTGCATCAATGAATGGT‐3′.

### 2.6 Western blot

RIPA lysis buffer (Beyotime, Shanghai, China), containing a cocktail of PMSF and proteinase inhibitors, was used for the extraction of total cellular proteins. The protein concentration was assessed using the CBA method. Following determination of protein concentration, 500 μg of protein was mixed with 5× SDS Loading Buffer in a 4:1 ratio and subjected to a 6‐min denaturation at 100°C in a metal bath. Each protein sample (20 μL) was subjected to 90‐min 10% SDS‐PAGE electrophoresis, and the proteins were then transferred onto a PVDF membrane. The membrane was blocked at room temperature for 1 h with 5% skim milk, followed by an overnight incubation with primary antibodies at 4°C. The following day, secondary antibodies were applied at room temperature for 1 h. The ECL exposure solution was evenly applied to the membrane, and protein bands were detected using a nucleic acid and protein gel imaging system (Bio‐Rad Laboratories, Hercules, CA, USA). Band intensities were measured using ImageJ software. Primary antibodies, including NLRP3 (A24294), ASC (A22555), Caspase‐1 (A0964), JAK2 (A19629), p‐JAK2 (AP0917), STAT3 (A22434), p‐STAT3 (AP0715), and GAPDH (A19056), were acquired from Abcam (Shanghai, China); JAK1 (A11963), STAT1 (A19563), STAT2 (A14995), p‐STAT1 (AP0054) were acquired from ABclonal (Wuhan, China); JAK3 (251322), p‐JAK3 (340809) were acquired from ZEN‐BIOSCIENCE (Chengdu, China); p‐JAK1 (AF2012), p‐STAT2 (AF8127) were acquired from Affinity (Liyang, China).

### 2.7 Preparation of faecal microbiota suspension

Samples were taken from three HUA‐induced ED rats, and treated with the optimal dose of the drug for 28 days. During this period, gentle abdominal massages were performed to facilitate defecation. The collected faeces were weighed and then homogenized with approximately five times the weight of physiological saline in a blender to ensure thorough mixing. The mixture was then filtered through a sieve to collect the suspension. The suspension was then transferred into a 10 mL centrifuge tube and centrifuged at 1200 r/min for 3 min. After removing the supernatant, an equal volume of physiological saline was added to the original suspension, followed by gentle inversion for mixing. This centrifugation and washing processes were repeated three times, and the supernatant was discarded, leaving purified faecal microorganisms as a precipitate. To the precipitate, three times its volume of physiological saline was added, and it was gently mixed to obtain the faecal bacterial solution.

### 2.8 Faecal microbiota transplantation (FMT)

Our experiment assigned six HUA‐induced ED rats into two groups: model group and faecal microbiota transplantation group (FMT group) (*n* = 3 per group). Rats were fasted but allowed to consume water at 6:00 PM the day before enema administration. The enema was administered twice the following day, at 8:00 AM and 4:00 PM. Before enema, gentle abdominal massage and stimulation were applied to eliminate residual faeces from the rats. Subsequently, the rats were anaesthetised with 2.1% isoflurane and fixed in a head‐down and tail‐up position. A lubricated enema needle was then gently inserted into the colon approximately 4–5 cm deep, and the faecal microbiota solution was slowly injected, with a retention time of 1 min to prevent leakage. After the enema procedure, the rats were released from the fixation and their mental states and activities were observed. If no abnormalities were detected, the rats were delivered to their cages and given unrestricted access to water. After the second enema each day, the rats were allowed free access to food until fasting resumed after 6:00 PM on the subsequent day, while water remained available. The entire experiment lasted for 5 days, with observations of the rats' activity and blood sample collection conducted on the 6th day.

### 2.9 Statistical analysis

For data analysis and visualization, we used GraphPad Prism 9.0. For the comparison of two groups, we used non‐parametric Student's *t*‐test. For comparisons among multiple groups, we employed one‐way ANOVA followed by post hoc testing. All data are expressed as mean ± standard deviation. A *p* < 0.05 was deemed statistically significant.

## 3 RESULTS

### 3.1 Impacts of linoleyl acetate on HUA‐induced ED rats

**FIGURE 1:**
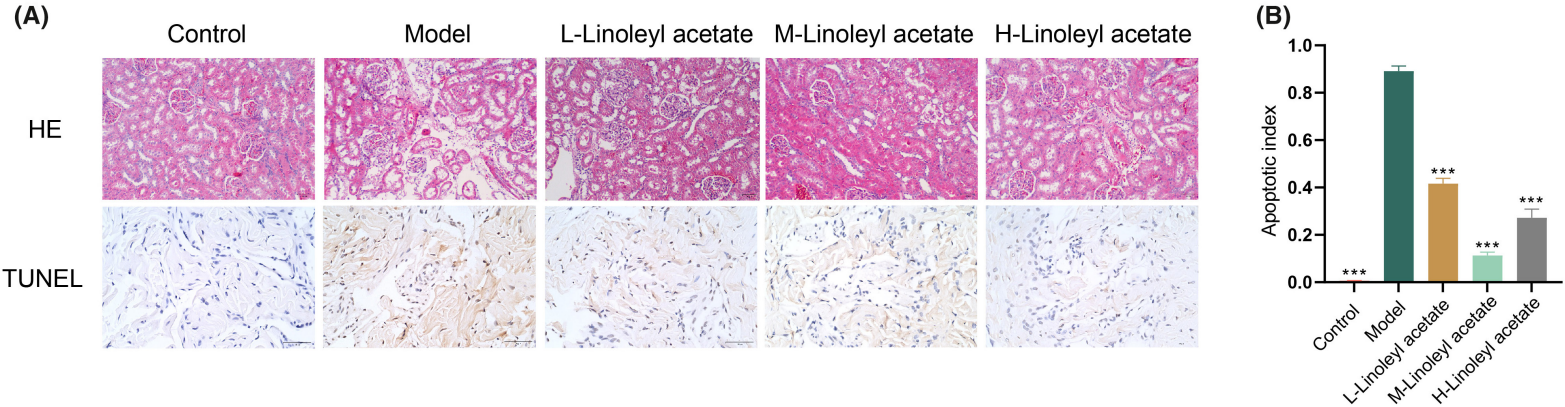
Impacts of linoleyl acetate on HUA‐induced ED in rats. (A) Histopathological examination (200× magnification) and TUNEL staining (400× magnification) outcomes for each group. (B) TUNEL assay was used to detect apoptosis index. Comparison with Model group, ***p < 0.001; *n* = 3.

To ascertain the impact of linoleyl acetate on HUA‐induced ED in rats, we established a rodent model. The results of HE staining and TUNEL staining are shown in Figure 1. After HE staining, the control group exhibited no discernible pathological alterations. Conversely, the model group showed renal glomerular atrophy, necrosis, structural deficiencies, swelling of renal tubular epithelial cells, vague morphological structures, cytoplasmic dissolution, partial epithelial cell necrosis, enlarged lumens, structural disruption, and internal shedding of epithelial cells. The low‐dose group manifested renal glomerular atrophy, structural deficiencies, swelling of renal tubular epithelial cells, cytoplasmic dissolution, enlarged lumens, and partial structural damage. The mid‐dose group demonstrated mild renal glomerular atrophy, closely arranged renal tubules, less epithelial cell degeneration, no observable fibrous tissue proliferation or inflammatory cell infiltration in the interstitium, and no other apparent pathological changes. The high‐dose group exhibited mild renal glomerular atrophy, slight water‐like degeneration of renal tubular epithelial cells, slight enlargement of the lumens, mild fibrous tissue proliferation in the interstitium, and no other conspicuous pathological changes. This suggested that the condition of HUA‐induced ED in rats was ameliorated, with the medium‐dose group yielding the most favourable results after administration. Using TUNEL staining, we detected a high level of apoptosis in penile tissue. Compared to the model group, the apoptosis indices significantly decreased in the low‐, medium‐, and high‐dose groups.

### 3.2 Linoleyl acetate inhibits the phosphorylation of JAK2 and STAT3 on HUA‐induced ED in rats

**FIGURE 2:**
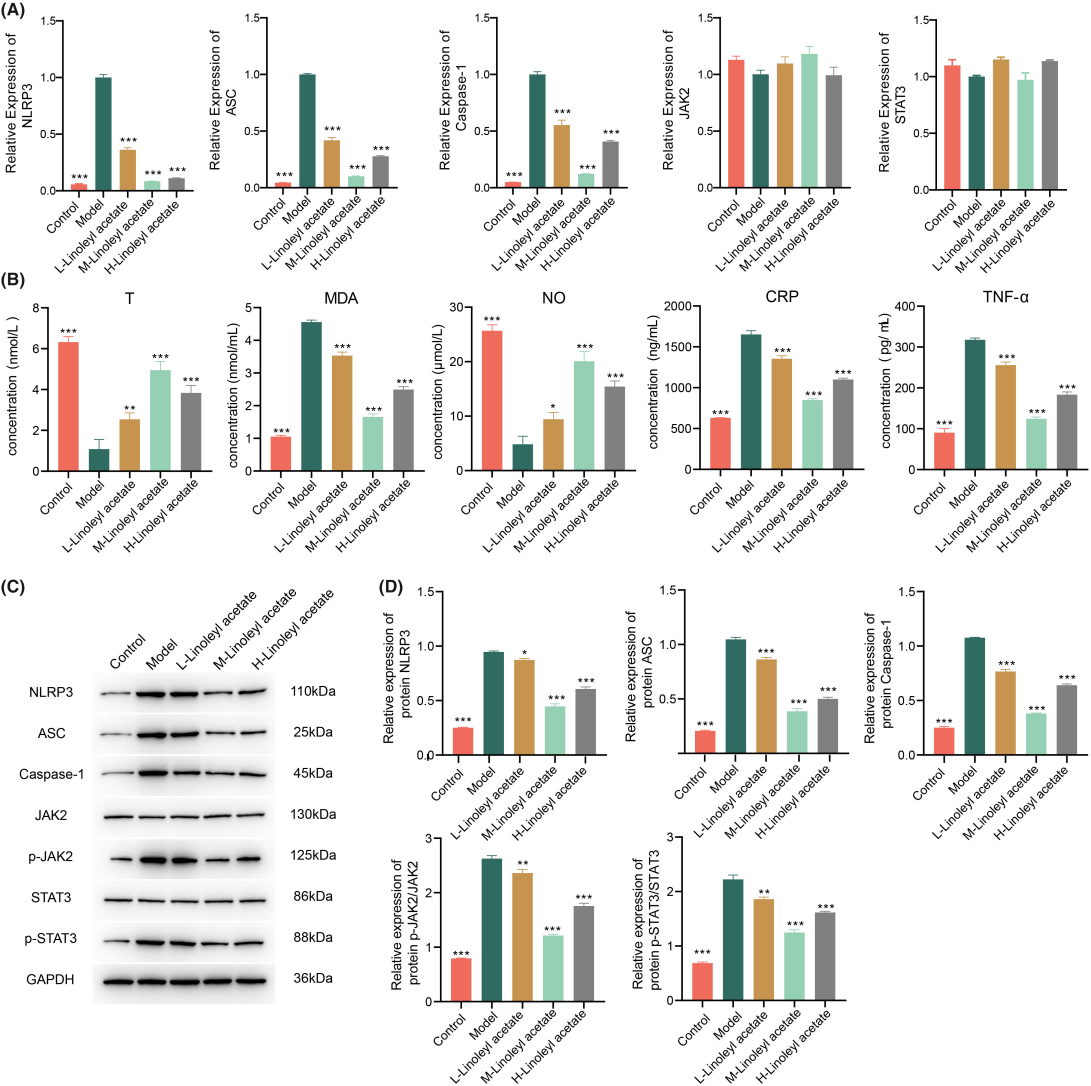
Effect of linoleyl acetate on expression in HUA‐induced ED rats. (A) qPCR analysis of alterations in NLRP3, ASC, Caspase‐1, JAK2, and STAT3 in the serum of HUA‐induced ED rats. (B) ELISA assessment of serum levels of T, MDA, NO, CRP, and TNF‐α in HUA‐induced ED rats. (C) Western blot analysis of expressions of NLRP3, ASC, Caspase‐1, JAK2, STAT3, p‐JAK2, and p‐STAT3 in various groups of HUA‐induced ED. (D) Protein expressions and ratios of NLRP3, ASC, Caspase‐1, p‐JAK2/JAK2, and p‐STAT3/STAT3 in the serum of rats with HUA‐induced ED. Comparison with Model group, *p < 0.05, **p < 0.01, ***p < 0.001; *n* = 3.

In contrast to the model group, the mRNA levels of NLRP3, ASC, and Caspase‐1 were significantly decreased (*p* < 0.01) in a dose‐dependent manner. There were no significant differences in JAK2 and STAT3 levels (Figure 2A). T and NO levels were significantly increased in HUA‐induced ED rats, while MDA, CRP, and TNF‐α levels were significantly decreased (Figure 2B). The protein levels of NLRP3, ASC, and Caspase‐1 exhibited significant reductions, while the ratios of p‐JAK2/JAK2 and p‐STAT3/STAT3 were also significantly decreased. Among the groups, the medium‐dose group demonstrated the most favourable outcomes (Figure 2C,D). In addition, the effects of linoleyl acetate on p‐JAK1/JAK1, p‐JAK3/JAK3, p‐STAT1/STAT1, p‐STAT2/STAT2 were tested, and no significant differences were found among all groups (Figure S1).

### 3.3 Impacts of FMT following linoleyl acetate treatment on HUA‐induced ED

**FIGURE 3:**
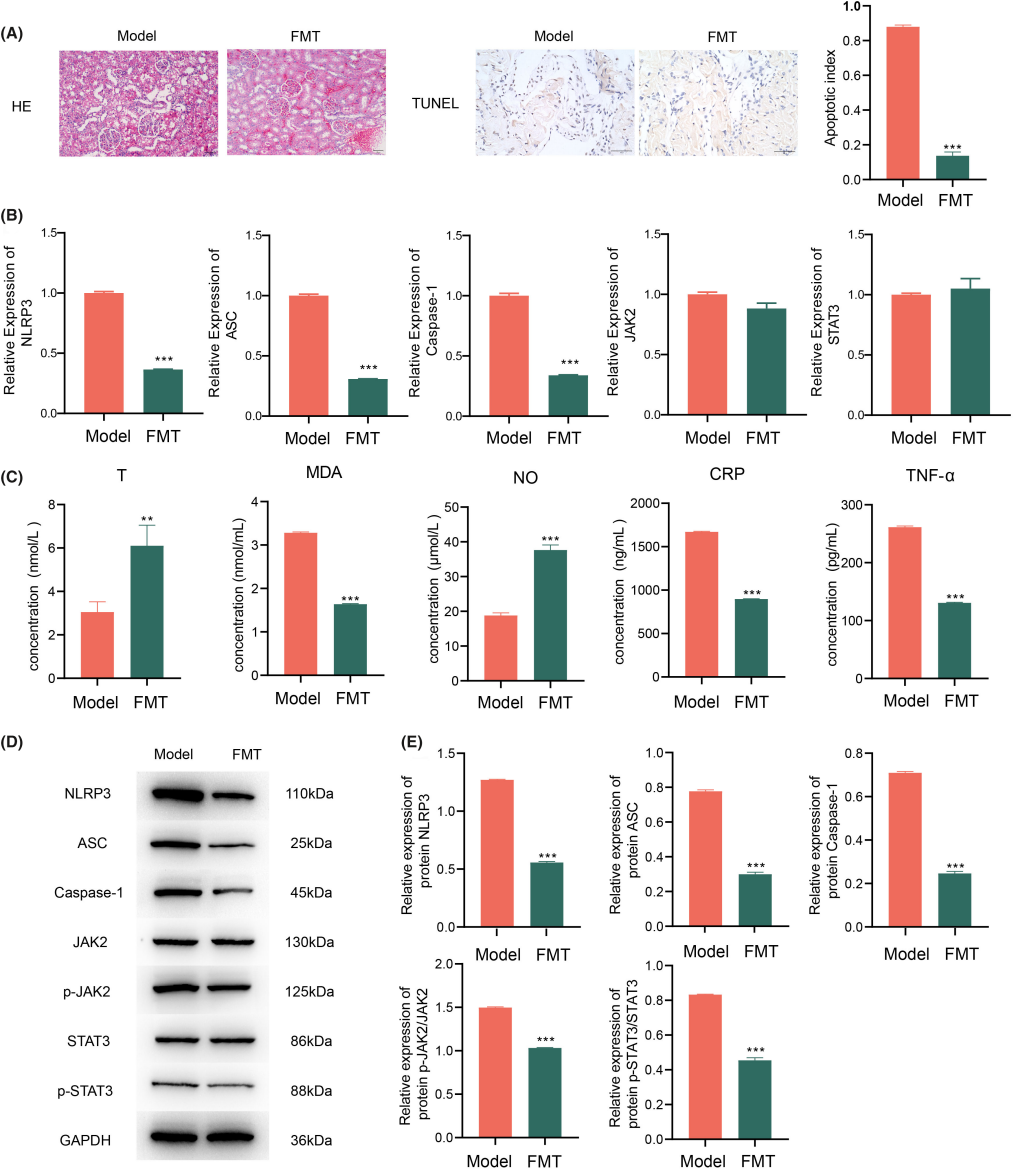
Impacts of FMT following linoleyl acetate treatment on HUA‐induced ED. (A) Assessment of staining conditions and apoptotic index via HE (200×) and TUNEL (400×) in each group. (B) qPCR analysis to examine alterations in NLRP3, ASC, Caspase‐1, JAK2, and STAT3 in the serum of HUA‐induced ED rats. (C) ELISA measurements of the levels of T, MDA, NO, CRP, and TNF‐α in the serum of HUA‐induced ED rats. (D) Western blot analysis to investigate the expressions of NLRP3, ASC, Caspase‐1, JAK2, STAT3, p‐JAK2, and p‐STAT3 in various groups of HUA‐induced ED. (E) Protein expressions and ratios of NLRP3, ASC, Caspase‐1, p‐JAK2/JAK2, and p‐STAT3/STAT3 in the serum of rats with HUA‐induced ED. Statistical significance was determined by comparing with Model group, where **p < 0.01; ***p < 0.001; *n* = 3.

FMT was performed after treatment with linoleyl acetate in medium‐dose group. HE staining revealed atrophy and necrosis of the renal glomeruli, structural defects, swelling of renal tubular epithelial cells, cytoplasmic dissolution, enlargement of lumens, structural damage, and internal shedding of epithelial cells, accompanied by mild interstitial fibrosis in model group. After faecal transplantation, the renal glomeruli exhibited mild atrophy, renal tubular epithelial cells displayed slight vacuolar degeneration, with only a small number of epithelial cells shed into the lumen, and no other notable pathological changes were observed (Figure 3A). TUNEL staining detected a high level of apoptosis in the penile tissue in the model group, while the FMT group showed a significant reduction in the apoptosis index (Figure 3A), demonstrating a dose‐dependent improvement in HUA‐induced ED for FMT group. Concurrent with linoleyl acetate treatment, compared to model group, levels of NLRP3, ASC, and Caspase‐1 were significantly decreased (*p* < 0.01), whereas JAK2 and STAT3 levels were not significantly different (Figure 3B). T and NO significantly increased in HUA‐induced ED rats, while MDA, CRP, and TNF‐α significantly decreased (Figure 3C), indicating that linoleyl acetate improved HUA‐induced ED by reducing inflammatory and oxidative stress reactions. Furthermore, there was a notable decrease in the protein expression of NLRP3, ASC, and Caspase‐1, along with significant reductions in the ratios of p‐JAK2/JAK2 and p‐STAT3/STAT3 (Figure 3D,E). However, there was no significant difference between p‐JAK1/JAK1, p‐JAK3/JAK3, p‐STAT1/STAT1, p‐STAT2/STAT2 (Figure S2).

### 3.4 Impacts of mandenol on HUA‐induced ED rats

**FIGURE 4:**
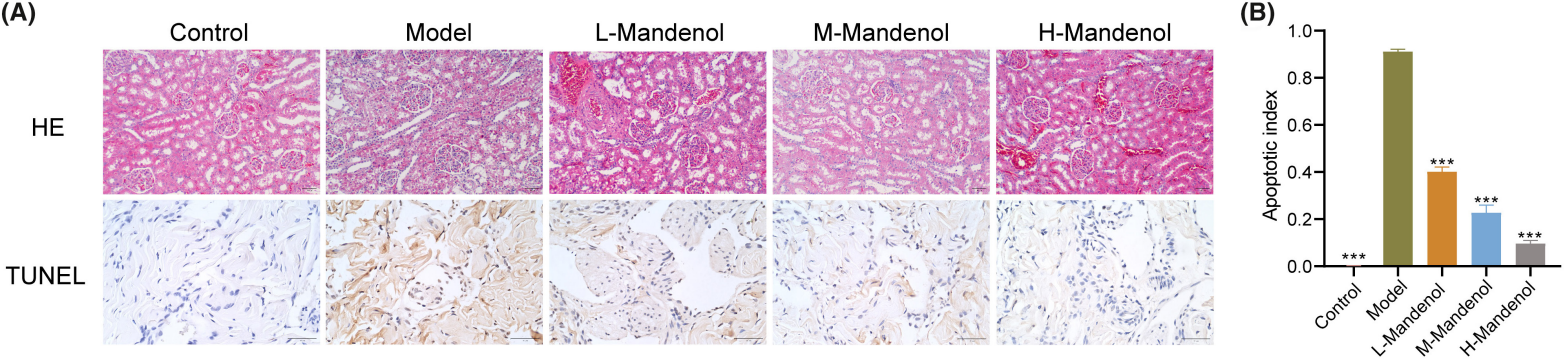
Impacts of mandenol on HUA‐induced ED in rats. (A) Histopathological examination (200×) and TUNEL staining (400×) outcomes for each group. (B) TUNEL assay was used to detect apoptosis index. Comparison with Model group, ***p < 0.001; *n* = 3.

To delve deeper into the potential of mandenol in ameliorating HUA‐induced ED, we established a rat model and administered mandenol via gastric lavage. The histopathological examination of rat kidney tissues using HE staining revealed negligible pathological changes in the control group. In contrast, the model group exhibited glomerular atrophy, necrosis, structural deficiencies, epithelial cell swelling in renal tubules, vague morphological structures, cytoplasmic dissolution, epithelial cell shedding in tubular lumens, and slight fibrous proliferation around the blood vessels. The low‐dose group displayed glomerular atrophy, epithelial cell swelling in renal tubules, cytoplasmic dissolution, epithelial cell shedding in tubular lumens, and noticeable congestion around blood vessels with slight fibrous proliferation in the surrounding areas. The medium‐dose group exhibited glomerular atrophy, partial swelling of epithelial cells in the renal tubules, shedding of epithelial cells in the tubular lumen, and pronounced vascular congestion. The high‐dose group showed mild glomerular atrophy, minimal epithelial cell degeneration, significant vascular congestion, and no other discernible pathological changes (Figure 4A). These findings suggested that mandenol treatment was capable of ameliorating HUA‐induced ED in rats in a dose‐dependent manner, and that the high‐dose group displayed the optimal outcome. TUNEL staining analysis indicated increased levels of apoptosis in penile tissue cells in the model group. Compared to the model group, the low‐, medium‐, and high‐dose groups showed significantly reduced apoptotic indices (Figure 4A,B).

### 3.5 Mandenol inhibits the phosphorylation of JAK2 and STAT3 on HUA‐induced ED in rats

**FIGURE 5:**
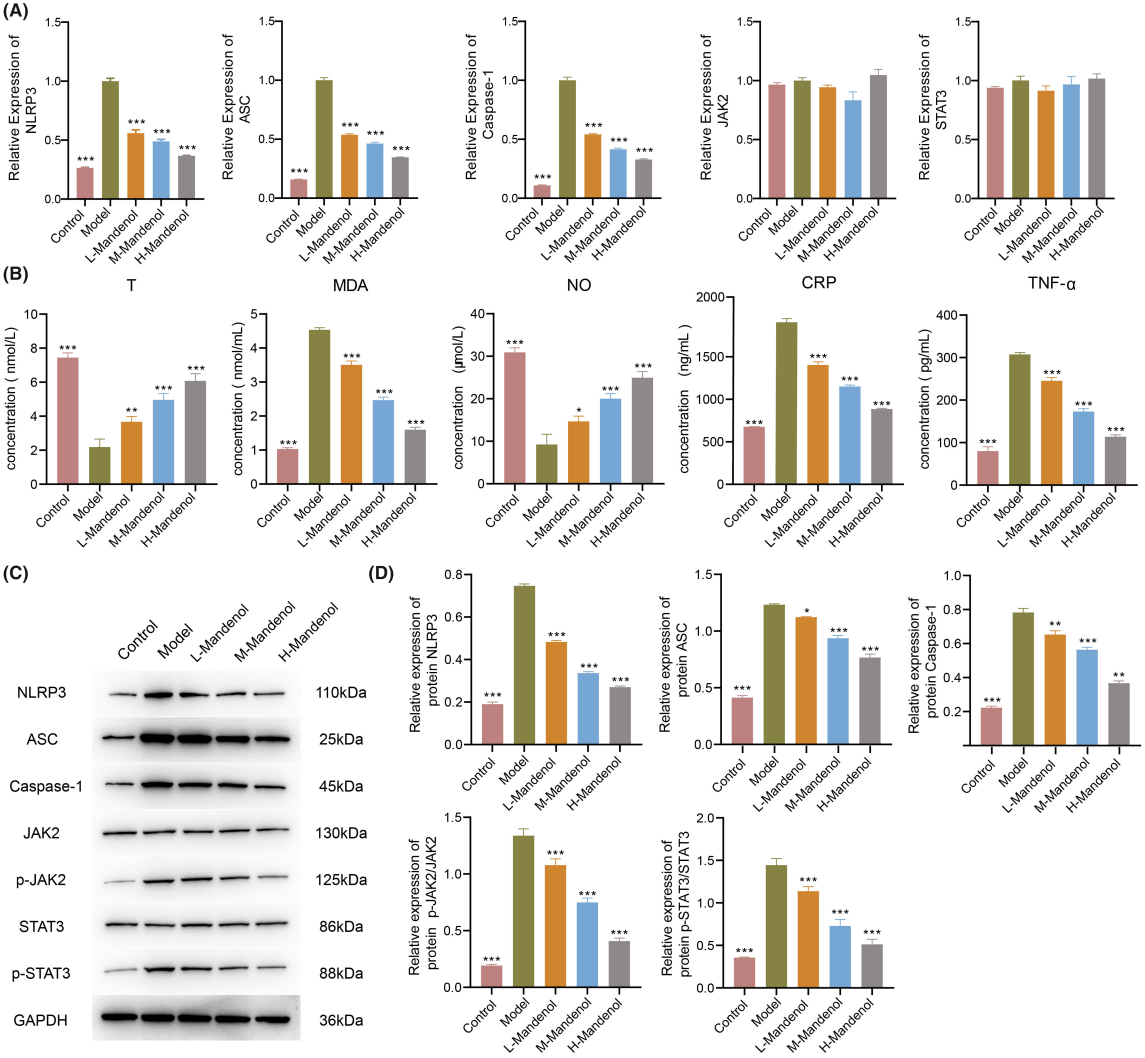
Effect of mandenol on expression in HUA‐induced ED rats. (A) qPCR examination of alterations in NLRP3, ASC, Caspase‐1, JAK2, and STAT3 in the serum of HUA‐induced ED rats. (B) ELISA analysis of serum levels of T, MDA, NO, CRP, and TNF‐α in HUA‐induced ED rats. (C) Western blot assessment of expressions of NLRP3, ASC, Caspase‐1, JAK2, STAT3, p‐JAK2, and p‐STAT3 in different groups of HUA‐induced ED rats. (D) Protein expressions and ratios of NLRP3, ASC, Caspase‐1, p‐JAK2/JAK2, and p‐STAT3/STAT3 in the serum of rats with HUA‐induced ED. Statistical comparisons were made against model group, where *p < 0.05; **p < 0.01; ***p < 0.001; *n* = 3.

Compared to the model group, mandenol treatment resulted in a substantial decrease in the levels of NLRP3, ASC, and Caspase‐1 (*p* < 0.01), with no significant differences observed in JAK2 and STAT3 (Figure 5A). T and NO levels significantly increased in HUA‐induced ED rats treated with mandenol, whereas MDA, CRP, and TNF‐α levels markedly decreased (Figure 5B). Western blot results indicated a significant reduction in the protein expression of NLRP3, ASC, and Caspase‐1, along with a marked decrease in the p‐JAK2/JAK2 and p‐STAT3/STAT3 ratios, with the high‐dose group showing the most favourable effect (Figure 5C,D). However, no significant differences were observed in p‐JAK1/JAK1, p‐JAK3/JAK3, p‐STAT1/STAT1, and p‐STAT2/STAT2 ratios (Figure S3). This implies that mandenol relieves HUA‐induced ED by reducing inflammation, oxidative stress, and JAK–STAT signalling pathway activity.

### 3.6 Impacts of FMT following mandenol treatment on HUA‐induced ED

**FIGURE 6:**
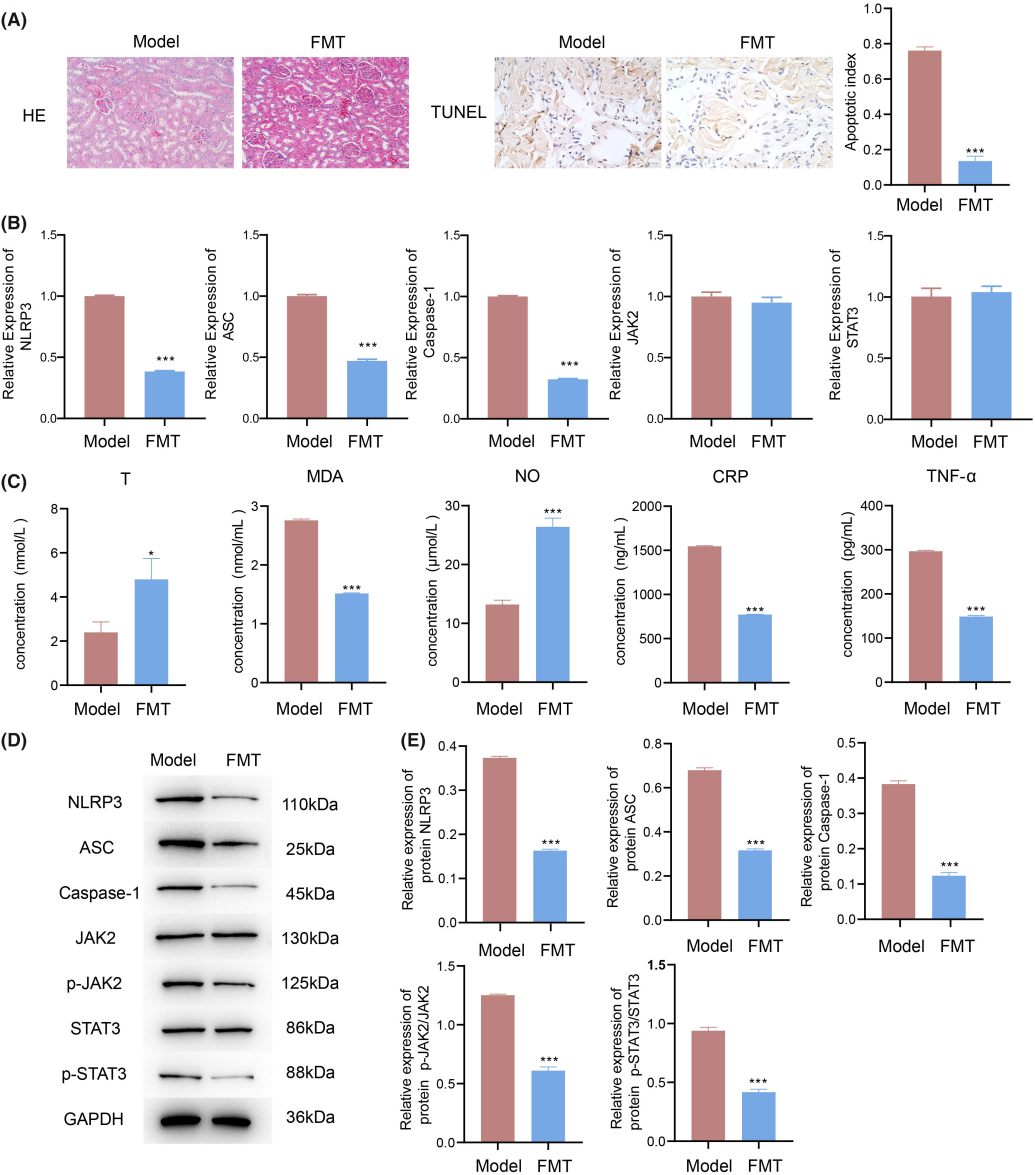
Post‐mandenol treatment, FMT reduces oxidative stress response and inflammatory reaction in HUA‐induced ED rats. (A) Evaluation of staining patterns and apoptosis index in various groups: Examination of HE (200×) and TUNEL (400×) staining outcomes, along with the assessment of the apoptosis index. (B) Analysis of serum changes in NLRP3, ASC, Caspase‐1, JAK2, and STAT3 using qPCR in rats with HUA‐induced ED. (C) Quantification of T, MDA, NO, CRP, and TNF‐α levels in the serum of rats with HUA‐induced ED through ELISA. (D) Western blot investigation into expressions of NLRP3, ASC, Caspase‐1, JAK2, STAT3, p‐JAK2, and p‐STAT3 across different groups in HUA‐induced ED rats. (E) Assessment of protein expression levels and ratios of NLRP3, ASC, Caspase‐1, p‐JAK2/JAK2, and p‐STAT3/STAT3 in the serum of rats with HUA‐induced ED. Comparisons were made with Model group, where *p < 0.05; ***p < 0.001; *n* = 3.

Following high‐dose mandenol treatment, faecal transplantation was performed. HE staining revealed notable pathological alterations in the model group, including glomerular atrophy, necrosis, structural defects, hydropic changes in renal tubular epithelial cells, blurred morphological structures, cytoplasmic dissolution, enlarged lumens, and disrupted architecture with internal shedding of epithelial cells. In contrast, the faecal transplantation group exhibited glomerular atrophy, mild vacuolar degeneration in renal tubular epithelial cells, the minor shedding of epithelial cells into the lumen, and no apparent proliferation of fibrous tissue or infiltration of inflammatory cells into the interstitium (Figure 6A). TUNEL staining demonstrated a significant reduction in the apoptosis index of penile tissue in the FMT group compared to that in the model group, indicating an ameliorative effect on HUA‐induced ED (Figure 6A). qPCR analysis revealed a significant dose‐dependent decrease in NLRP3, ASC, and Caspase‐1 levels in the FMT group (*p* < 0.01), while no significant differences were observed in JAK2 and STAT3 (Figure 6B). In HUA‐induced ED rats of the FMT group, there was a significant increase in T and NO levels, alongside a substantial decrease in MDA, CRP, and TNF‐α levels (Figure 6C). Moreover, the protein expression of NLRP3, ASC, and Caspase‐1 showed significant reductions, along with markedly decreased ratios of p‐JAK2/JAK2 and p‐STAT3/STAT3 (Figure 6D,E). However, there was no significant difference between p‐JAK1/JAK1, p‐JAK3/JAK3, p‐STAT1/STAT1, p‐STAT2/STAT2 (Figure S4).

## 4 DISCUSSION

ED is a common male sexual disorder, often influenced by HUA, recognized as a significant contributing factor.^16^ The Chinese herbal decoction, QYHT, has been proven to reduce the occurrence of related complications such as impotence, with the key compounds linoleyl acetate and mandenol potentially effective as well. Linoleyl acetate, a naturally occurring compound, has been shown by Li et al.^17^ to exert anti‐inflammatory effects through various pathways. Specifically, Gamma–Linoleyl acetate has been demonstrated to reduce the production of inflammatory cytokines by inhibiting the NF‐κB signalling pathway. Mandenol can affect the TCA cycle by acting on phosphoenolpyruvate carboxykinase.^18^ However, their impact on HUA‐induced ED remains unclear. This study aimed to explore the effects of different concentrations of linoleyl acetate and mandenol on HUA‐induced ED.

As an important neurotransmitter, NO plays a critical role in the induction of priapism. Upon sexual stimulation, activated parasympathetic stimulation prompts vascular endothelial cells and spongiform nerves to produce NO.^19^ Meanwhile, T is the main androgen, and a key index of sex hormone metabolism, significantly contributes to the maturation of male sperm, maintenance of normal sexual desire, and reproductive function. A diminished T level stands as the main cause behind reproductive health problems such as male infertility and ED.^20^ Lyngdoh et al.^10^ have reported a positive correlation between blood uric acid levels and CRP, TNF‐α, interleukin (IL) expression, implying that HUA can stimulate monocytes, release various inflammatory cytokines, trigger systemic sterile inflammatory response, and play an important role in the apoptosis pathway. These results suggest an important role of HUA in promoting inflammation and apoptosis, which may be related to the development of ED and other diseases. In this study, treatment using linoleyl acetate and mandenol inhibited cell apoptosis in penile tissues, which significantly increased T and NO, but greatly decreased MDA, CRP, and TNF‐α, which is consistent with the results of previous studies. This suggests that linoleyl acetate and mandenol may have a positive effect on ED caused by HUA through multiple pathways to improve the expression levels of relevant biomarkers.

Uric acid has antioxidant and anti‐inflammatory effects, serving as a safeguard for vascular endothelial cells against oxidative stress and inflammatory agents. Normally, uric acid exists in a soluble urate form, with its synthesis and excretion intricately balanced to maintain a dynamic equilibrium. However, excessive consumption of purines, metabolic disorders or enzyme abnormalities can disrupt this balance, leading to increased serum uric acid levels. Once uric acid surpasses 408 μmol/L, it can precipitate sodium urate crystal formation, subsequently activating the NLRP3 inflammasome. The NLRP3 inflammasome is an important multicomponent assembly that comprising NLRP3, ASC and Caspase‐1, highly expressed in myeloid cells. This complex plays a key defensive role in the body's innate immunity and stress systems.^21^ As an intracellular pattern recognition receptor, NLRP3 can be activated by hyperuric acid or urate crystals, leading to the secretion of IL‐1β, IL‐18β and IL‐18, thereby contributing to the pathogenesis of diverse inflammatory diseases. It also detects pathogen‐associated molecular patterns (PAMPs) or host‐derived danger signal molecules (DAMPs), initiating inflammatory responses by recruiting and activating the pro‐inflammatory protease Caspase‐1. Once activated, Caspase‐1 cleaves the precursors of IL‐1 and IL‐18, producing mature cytokines that subsequently trigger a cascade of inflammatory responses.^22^ Research has demonstrated the activation of the NLRP3 inflammasome across different autoinflammatory and autoimmune conditions, including gout, rheumatoid arthritis, systemic lupus erythematosus, and more, indicating its crucial involvement in the onset and advancement of inflammatory disorders.^23^

The Janus kinase/signal transduction and transcription (JAK/STAT) pathway serves as the principal route for cytokine signal transduction, participating in the inflammatory processes of cytokine signalling. Additionally, JAK2 and STAT3 have been identified to exert beneficial and protective effects in modulating inflammatory responses.^24^, ^25^, ^26^ The overactive expression of STAT1 and STAT3, as well as the activated JAK–STAT signalling transduction, has been implicated in the progression of nephritis and the onset of proteinuria.^27^ Among these, JAK2 is regarded as a significant contributor to ED.^28^ Consequently, in clinical practice, the treatment of diabetes‐induced ED often involves the inhibition of JAK2 and the reduction of oxidative stress.^29^ However, there is limited research on regulating the JAK2/STAT3 pathway in rats with HUA‐induced ED. Linoleyl acetate possesses anti‐inflammatory properties.^30^ Mandenol, identified in a study by Zhang et al. on treating diabetic nephropathy with *Cornus officinalis*, has been proven to possess essential fatty acid properties with antibacterial and anti‐inflammatory characteristics.^31^, ^32^ Their research also suggested that mandenol could suppress inflammatory activity by reducing the expression of inducible nitric oxide synthase and cyclooxygenase‐2.^33^ In this study, we established a rat model of HUA to determine whether linoleyl acetate and mandenol could alleviate HUA‐induced ED damage by regulating NLRP3 inflammasome and JAK/STAT signalling. We found that the mRNA and protein expression levels of NLRP3, ASC and Caspase‐1 after linoleyl acetate and mandenol treatment were significantly reduced, and the phosphorylation of JAK2 and STAT3 was significantly inhibited, and the FMT results showed the similar results. However, linoleyl acetate and mandenol did not significantly affect the phosphorylation of JAK1, JAK3, STAT1 and STAT2. This could be due to the unique roles of each JAK.^34^ For example, Tofacitinib interfered with JAK–STAT signal transduction by competing with ATP for binding to the JAK kinase domain, and inhibited JAK1, JAK2 and JAK3. However, in vitro studies have shown that JAK1 and JAK3 were preferentially inhibited, with less effect on JAK2.^35^, ^36^ R723, an orally bioavailable small molecule, could effectively inhibit JAK2, but had minimal effect on the activity of JAK3, TYK2 and JAK1.^37^ The roles of STATs also vary. Previous studies have shown that cholesterol crystals activate STAT1, but have no effect on the phosphorylation of STAT2, STAT3, STAT4, STAT5 and STAT6.^38^ IFN‐β exhibits antifibrotic effects through STAT1 and STAT2 in CF TGF‐β1 treated cells, while STAT3 is not involved in this effect.^39^ This suggests that NLRP3 inflammasome and the JAK2/STAT3 signalling may be related to HUA‐induced ED, and that linoleyl acetate and mandenol may ameliorate the ED in rats by inhibiting the release of NLRP3 inflammasome and the expression of the JAK/STAT pathway.

Existing treatments for ED, such as sildenafil, while effective, might cause side effects such as headache, facial flushing, and visual disturbances.^40^, ^41^ Additionally, existing treatment methods primarily targeted vasodilation and overlooked the influence of gut microbiota on ED.^42^ Our study indicated that linoleyl acetate and mandenol, as the main components of the traditional Chinese medicine QYHT decoction, improved gut microbiota and ED by regulating the metabolites of gut microbiota. Our study validated this mechanism for the first time using FMT. Furthermore, our research suggested that linoleyl acetate and mandenol have multi‐target effects, not only improving vasodilation but also regulating the function of the nervous system, thereby providing a more comprehensive improvement for ED. However, this study also has certain limitations. First, this experiment only examined the effects of adding linoleyl acetate or mandenol separately, and the combined effect of linoleyl acetate + mandenol remains unclear. Second, the study was conducted only in animal models, necessitating further validation in human clinical trials to confirm these findings. In future research, the combined effect of linoleyl acetate + mandenol could be investigated, and metabolomics testing could be included to explore changes in metabolites.

Taken together, in this study, using an HUA‐induced ED rat model, we found that linoleyl acetate and mandenol mitigate HUA‐induced ED damage by inhibiting the NLRP3 inflammasome and JAK/STAT signalling pathways. Our results demonstrate that these compounds effectively reduce the expression levels of NLRP3, ASC, and Caspase‐1, while inhibiting the phosphorylation of JAK2 and STAT3. These findings suggest that the NLRP3 inflammasome and JAK/STAT signalling pathways may contribute to the development of HUA‐induced ED. This suggests a promising therapeutic approach for treating HUA‐induced ED, providing a theoretical basis for future treatment strategies.

## AUTHOR CONTRIBUTIONS

**Pingyu Ge:** Conceptualization (equal); writing – original draft (equal). **Hong Xie:** Writing – original draft (equal). **Yinxue Guo:** Methodology (equal). **Hang Jin:** Software (lead). **Lan Chen:** Formal analysis (equal). **Zhichao Chen:** Formal analysis (equal). **Yan Liu:** Conceptualization (equal); writing – review and editing (lead).

## FUNDING INFORMATION

This study was supported by the Science and Technology Foundation of Guizhou Province (Guizhou Science and Technology Foundation, ZK [2021] General 507).

## CONFLICT OF INTEREST STATEMENT

The authors declare they have no conflict of interest.

## Supporting information


Figure S1.

## Data Availability

All data generated or analysed during this study are included in this article.
