# Supplementary material for: Linoleyl acetate and mandenol alleviate HUA‐induced ED via NLRP3 inflammasome and JAK2/STAT3 signalling conduction in rats
Source: J Cell Mol Med. 2024 Sep 8;28(17):e70075. doi: 10.1111/jcmm.70075 (PMC11381191; doi:10.1111/jcmm.70075)
Supplement: Supplementary file 1 — Figure S1. [file JCMM-28-e70075-s001.docx]

**Supplementary material**


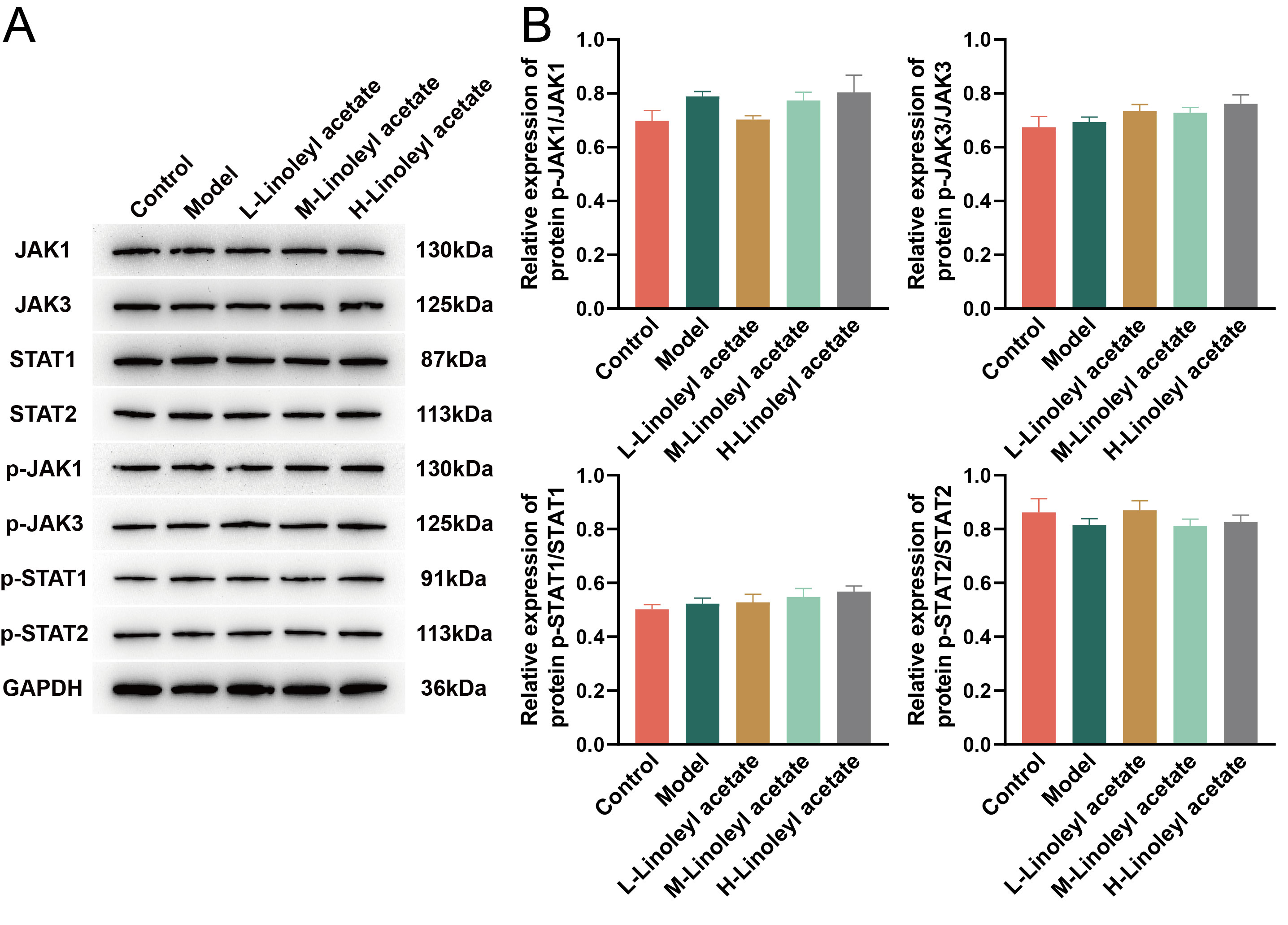


**Figure S1 Western blot analysis was performed to investigate the effects of linoleyl acetate on protein expression in different groups of HUA-induced ED rats.** A. Representative Western blot bands showing protein expression of JAK1, JAK3, STAT1, STAT2, p-JAK1, p-JAK3, p-STAT1, and p-STAT2 in different groups of HUA-induced ED rats. B. Quantitative analysis of p-JAK1/JAK1, p-JAK3/JAK3, p-STAT1/STAT1, and p-STAT2/STAT2 ratios in different groups of HUA-induced ED rats.


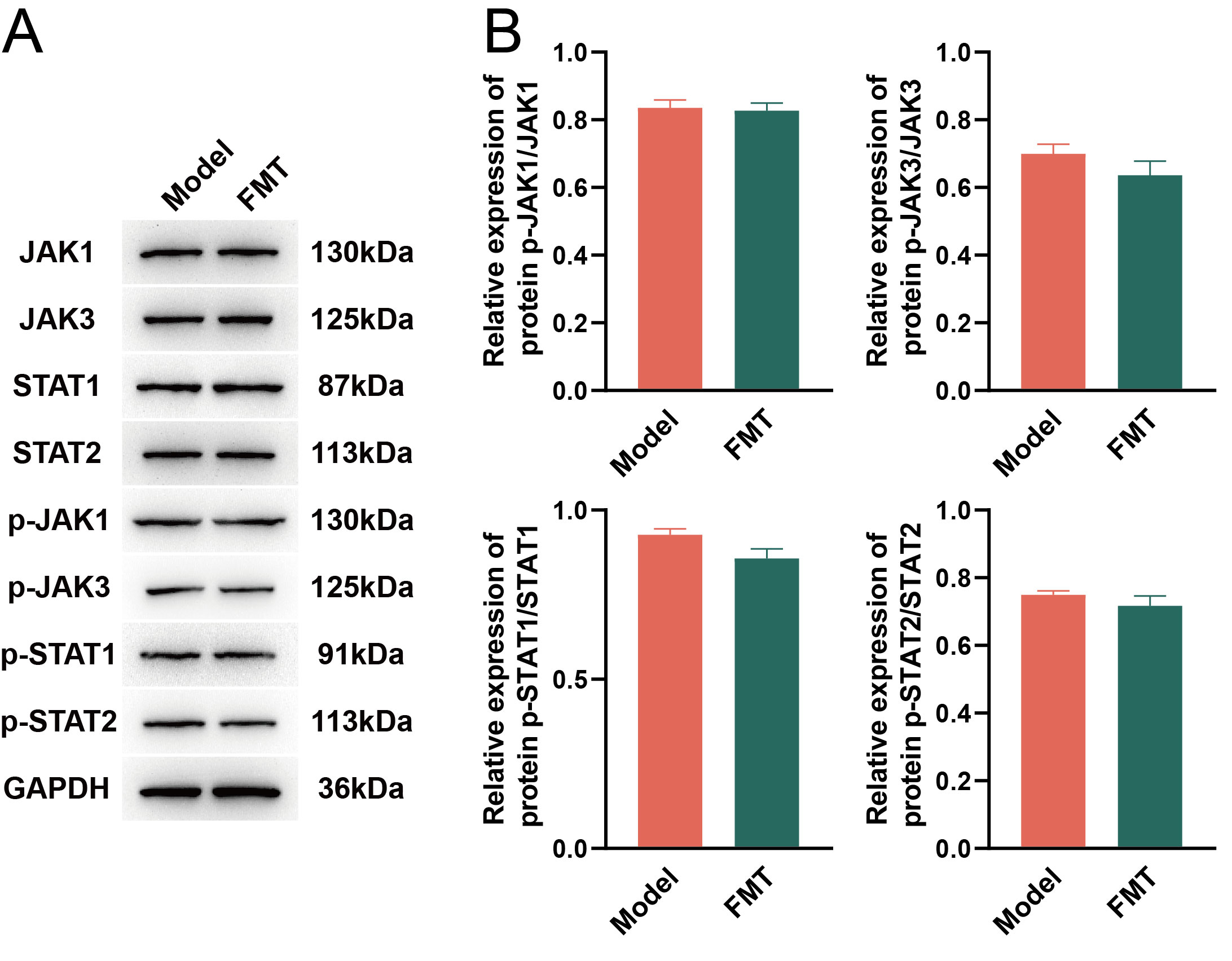


**Figure S2 Effect of FMT on Protein Expression in linoleyl Acetate-Treated HUA-Induced ED Rats.** A. Representative Western Blot Bands Showing Protein Expression of JAK1, JAK3, STAT1, STAT2, p-JAK1, p-JAK3, p-STAT1, and p-STAT2 in Different Groups of HUA-Induced ED Rats. B. Quantitative Analysis of p-JAK1/JAK1, p-JAK3/JAK3, p-STAT1/STAT1, and p-STAT2/STAT2 Ratios in Different Groups of HUA-induced ED Rats.


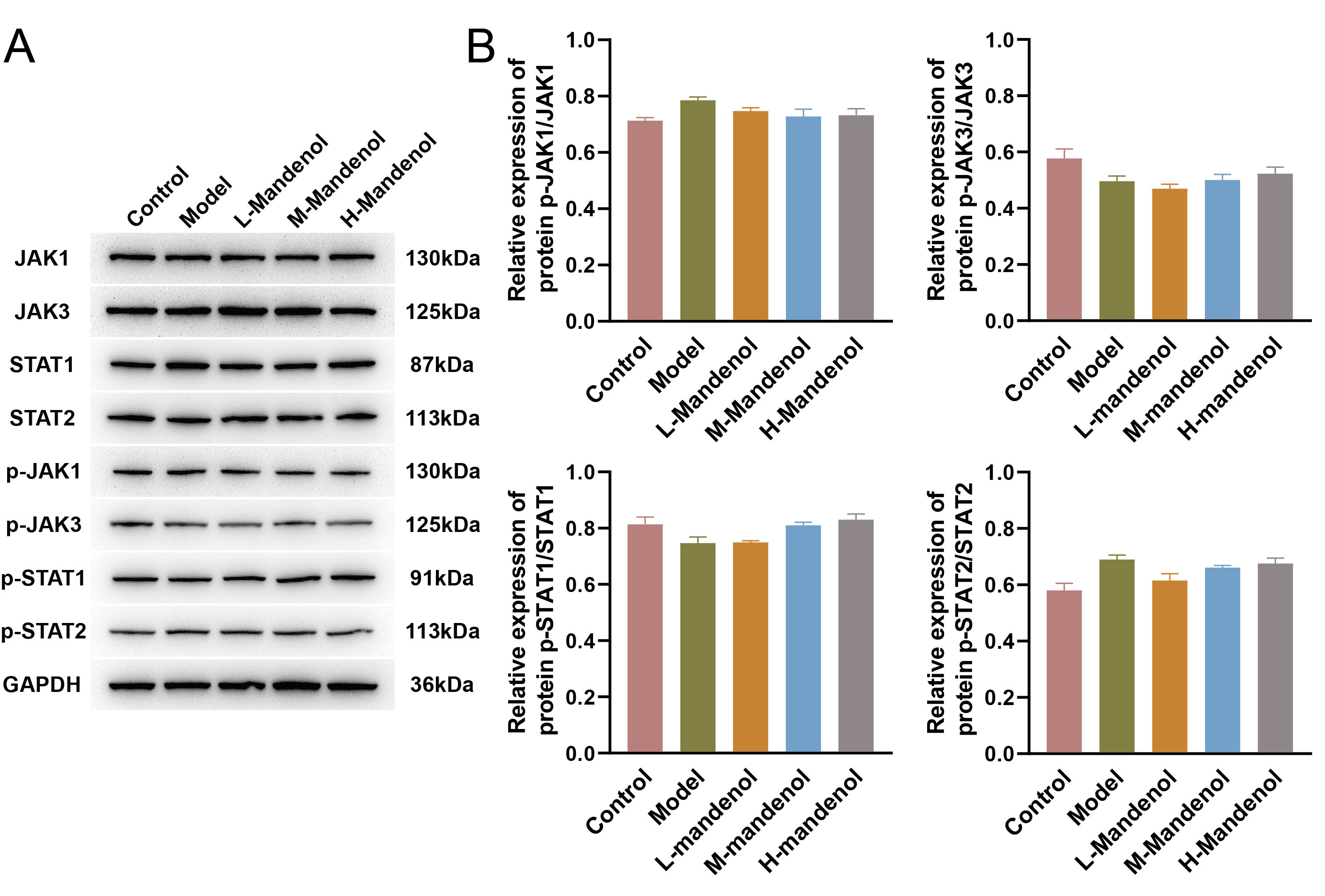


**Figure S3 Western blot analysis was performed to investigate the effects of** **mandenol on protein expression in different groups of HUA-induced ED rats.** A. Representative Western blot bands showing protein expression of JAK1, JAK3, STAT1, STAT2, p-JAK1, p-JAK3, p-STAT1, and p-STAT2 in different groups of HUA-induced ED rats. B. Quantitative analysis of p-JAK1/JAK1, p-JAK3/JAK3, p-STAT1/STAT1, and p-STAT2/STAT2 ratios in different groups of HUA-induced ED rats.


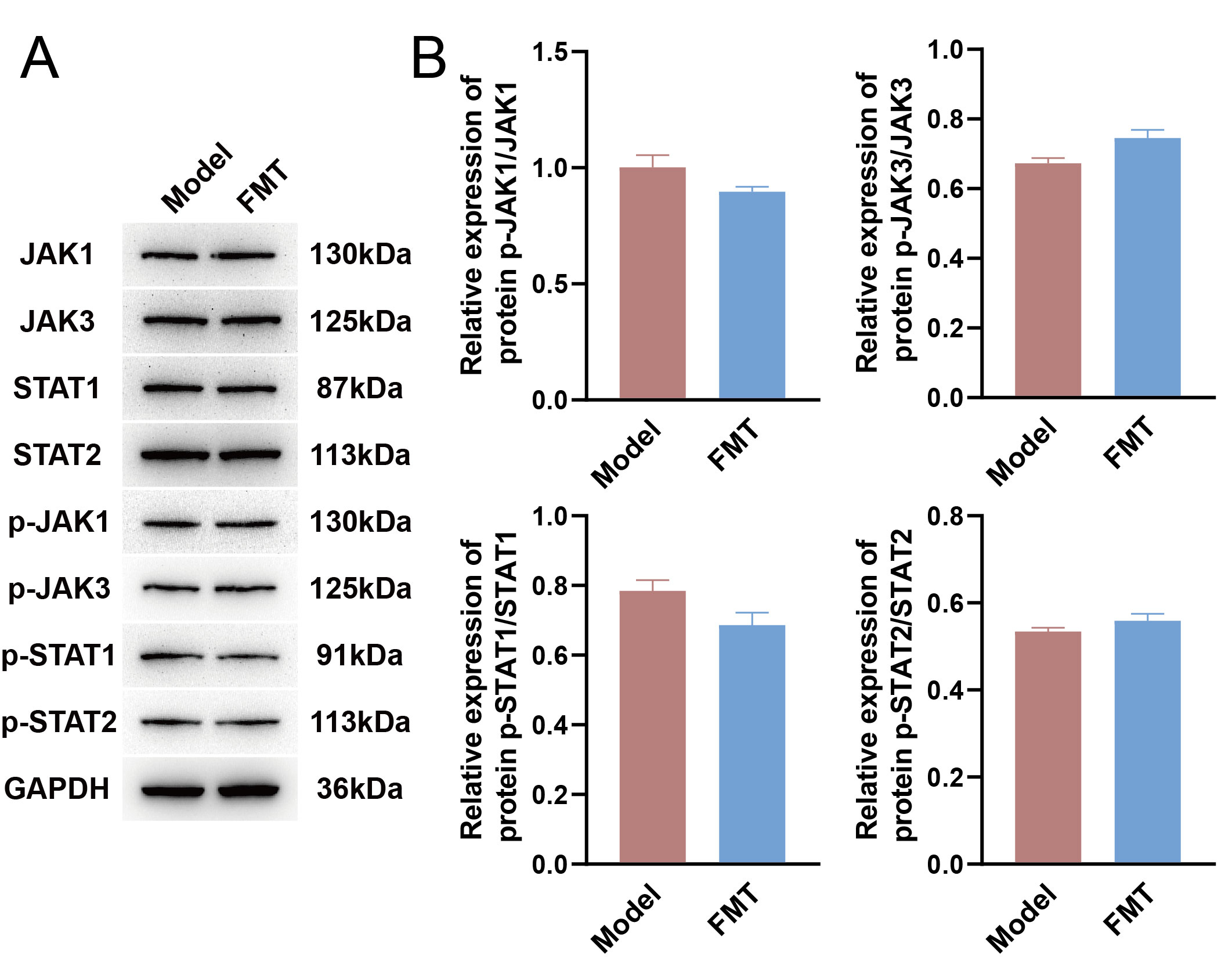


**Figure S4 Effect of FMT on Protein Expression in mandenol-Treated HUA-Induced ED Rats.** A. Representative Western Blot Bands Showing Protein Expression of JAK1, JAK3, STAT1, STAT2, p-JAK1, p-JAK3, p-STAT1, and p-STAT2 in Different Groups of HUA-Induced ED Rats. B. Quantitative Analysis of p-JAK1/JAK1, p-JAK3/JAK3, p-STAT1/STAT1, and p-STAT2/STAT2 Ratios in Different Groups of HUA-induced ED Rats.
